# Can apneic oxygen insufflation become a novel lung protective ventilation strategy? A randomized, controlled, blinded, single center clinical trial

**DOI:** 10.1186/s12871-018-0652-z

**Published:** 2018-12-11

**Authors:** Yongtao Gao, Zhi Wang, Feng Jiang, Jie Gao, Yujia Li, Siyuan Liu

**Affiliations:** 1 Affiliated Hospital of Nantong University, No. 20 Xishi Road, 226001 Nantong City, Jiangsu Province People’s Republic of China; 2 Medical College of Nantong University, Nantong, People’s Republic of China

**Keywords:** One lung ventilation, Apneic oxygen insufflation, Oxygenation index, Inflammatory response, Oxidative stress, Bronchoalveolar lavage fluid

## Abstract

**Objective:**

The aim of this study was to determine whether a AOI strategy on non-ventilated lung could reduce the regional and systemic proinflammatory cytokine and oxidative stress response associated with esophagectomy, and to evaluate whether AOI can be used as a novel lung protective ventilation strategy. Its impact on oxygenation after OLV, surfactant protein A, B, C (SP-A, B, C), postoperative hospital stay and postoperative pulmonary complications (PPCs) was also evaluated.

**Methods:**

Fifty-four adults (ASA II-III) undergoing esophagectomy with OLV were enrolled in the study. Patients were randomly assigned into 2 groups: control group (group C) and treated group (group T). Group C was treated with traditional OLV mode,while group T was given AOI of 5 L/min oxygen on the non-ventilated lung immediately at the beginning of OLV. Arterial blood gas was analyzed before and after OLV. A bronchoalveolar lavage(BAL) was performed after OLV on the non-ventilated lung. Proinflammatory cytokine, oxidative stress markers(TNF-α, NF-κB,sICAM-1,IL-6,IL-10,SOD,MDA) and SP-A, B, C were analyzed in serum and BALF as the primary endpoint.The clinical outcome determined by PPCs was assessed as the secondary endpoint.

**Results:**

Patients with AOI had better oxygenation in the recovery period, oxygenation index(OI) (394[367–426] and 478[440–497]mmHg, respectively) of group T at T_2_ and T_3_ were significantly higher than those (332[206–434] and 437[331–512]mmHg, respectively) of group C. OLV resulted in an increase in the measured inflammatory markers in both groups, however, the increase of inflammatory markers upon OLV in the group C was significantly higher than those of group T. OLV resulted in an increase in the measured SP-A, B, C in serum of both groups. However, the levels of SP-A, B, C of group T were lower than those of group C in serum after OLV, and the results in BALF were the opposite. The BALF levels of SOD(23.88[14.70–33.93]U/ml) of group T were higher than those(15.99[10.33–24.16] U/ml) of group C, while the levels of MDA in both serum and BALF of group T(8.60[4.14–9.85] and 1.88[1.33–3.08]nmol/ml, respectively) were all lower than those of group C (11.10[6.57–13.75] and 1.280[1.01–1.83]nmol/ml) after OLV. There was no statistical difference between the two groups in terms of postoperative hospital stay and the incidence of PPCs.

**Conclusion:**

AOI on non-ventilated lung during OLV can improve the oxygenation function after OLV, relieve the inflammatory and oxidative stress response in the systemic and non-ventilated lung after OLV associated with esophagectomy.

**Trial registration:**

ChiCTR-IOR-17011037. Registered on 31 March 2017.

## Introduction

As a non-physiological ventilation mode, OLV causes hypoxemia, acute lung injury (ALI) and even acute respiratory distress syndrome (ARDS). At present, ALI and ARDS have become one of the major causes of postoperative death in thoracic patients [1]. Factors such as the collapse/recruitment of non-ventilated lung, decreased oxygenation function, inflammatory reaction and lung injury caused by mechanical ventilation during OLV can all lead to pathophysiological changes of patients [2].The non-ventilated lung in the thoracic surgery during OLV do not have oxygenation but still have blood supply result that the value of Qs/Qt increase [3]. Hypoxemia is the most important factor affecting the physiology of patients [4], which is the key to the serious complications during and after operation, and also the key to the success of thoracic surgery.An investigation has shown that the incidence of hypoxemia caused by OLV is about 9–27% [5]. Therefore, it is especially important to take concrete measures to alleviate lung injury caused by OLV.

It has been reported that the selective oxygen supply of non-ventilated lungs at a flow rate of 5 L/min through the fiberoptic bronchoscope significantly improves oxygenation while not affects surgical operations [6], this method is called apneic oxygen insufflation(AOI).Literature [6, 7] and our previous studies [8] have shown that AOI with oxygen flow in 5 L/min can improve arterial oxygenation function and reduce the intrapulmonary shunt rate during OLV, and moderating lung collapse with highest satisfaction of surgeons. However, it has not been reported the effects of AOI on oxygenation function, inflammation and oxygenation stress response after OLV and long-term clinical outcome, we therefore were interested to explore the potential benefits of AOI for patients undergoing thoracic surgery.

The aim of this study was to determine whether a AOI strategy on non-ventilated lung could reduce the regional and systemic proinflammatory cytokine and oxidative stress response associated with esophagectomy, and to evaluate whether AOI can be used as a novel lung protective ventilation strategy.Its impact on oxygenation after OLV, surfactant protein A, B, C, postoperative hospital stay and postoperative pulmonary complications was also evaluated.

## Methods

### Research agreements

This randomized, controlled, clinical trial was completed by Affiliated Hospital of Nantong University. This study has been approved by the Ethic Committee of Affiliated Hospital of Nantong University (Chairperson Prof. Zhangtao) on 19 September 2016, and registered on Chinese Clinical Trial Registry (registration number: ChiCTR-IOR-17011037, Registered on 31 Match 2017). All the patients have signed informed consents, and the ethical processes of the study conformed to the “Declaration of Helsinki”.

### Case selection

Eligible patients met the following criteria: (1) aged 50 to 75 years old, (2) planned esophagectomy for esophageal and cardia cancer, (3) the American Society of Anesthesiologists status II to III, and (4) the estimated duration of the operation is 1 to 4 h. Exclusion criteria: (1) patients with previous history of thoracic surgery, (2) patients with liver and renal insufficiency, or left ventricular dysfunction, (3) patients with arrhythmia affecting hemodynamic stability, (4) patients with asthma, severe emphysema (residual volume > 75%), (5) preoperative corticosteroid treatment during the month before inclusion or a preoperative acute infection suspected because of a temperature greater than 38 °C or less than 36 °C, leukocyte count greater than 10 × 10^9^ or less than 4 × 10^9^, pulmonary infection or any new pulmonary infiltrate on the systematic chest radiograph, (6) secondary surgery, and (7) patients enrolled in other clinical test within 3 months.

### Randomization and methods of AOI

The random numbers automatically generated by the computer according to the random number table were placed in the envelopes and sealed. Patients were randomly divided into two groups by random figure table: control group (group C) and treated group (group T). Self-made AOI device includes oxygen inhaler on the wall, humidification bottle, single use drainage tube, and 10FR suction tube. The 10FR suction tube was inserted through the trachea to a distance of 1–2 cm below the carina of the non-ventilated side [7]. The lumen of the non-ventilated side DLT must be completely open, so that the air between the suction catheter and the DLT can freely circulate.

### Methods of anesthesia

All patients in the groups underwent right internal jugular vein catheterization with regional anesthesia. Invasive arterial blood pressure(IBP) was monitored by radial artery puncture and catheterization. All patients of two groups adopted a unified anesthetic induction and maintenance scheme based on total intravenous anesthesia, and maintained a proper depth of anesthesia, i.e. BIS ranges from 40 to 60.

Left-sided double lumen tube(DLT) (Weili Company. China, Fr37 for male and Fr35 for female) were inserted. A20–4.2 type fiberoptic bronchoscope (Medward, China) was used to confirm the position of the DLT before and after the patient was placed in the lateral position.Mechanical ventilation was performed with tidal volume(VT) of 8-10 ml/kg and FiO2 0.5 during two-lung ventilation(TLV), and 5-6 ml/kg and 1.0 during OLV, combined with some recruitment maneuvers every 30 min, both with oxygen flow 2 L/min, I:E = 1:2, positive end expiratory pressure: 0cmH2O (ZEEP), respiratory rate 11-12 bpm for TLV and 14-16 bpm for OLV. Ventilatory frequency was set to maintain P_ET_CO_2_ at 25-40 mmHg. OLV was performed prior to surgical thoracotomy and peak airway pressure was maintained below 25 cmH_2_O during OLV. Group C was treated with traditional OLV mode, while group T was given AOI of 5 L/min oxygen on the non-ventilated lung immediately at the beginning of OLV.

### Methods of Bronchoalveolar lavage

Bronchoalveolar lavage was performed on the non-ventilated lung after OLV. Lavage position is recommended to choose the right middle lobe or left lung lingual segment [9].The tip of the fiberoptic bronchoscope was wedged tightly to the bronchial opening of the B5 segment of the right lung (intraoperative left lung ventilation) or the tongue segment of the left lung(intraoperative right lung ventilated), and the biopsy hole was rapidly injected with sterile saline at 37 °C, a total of 50 ml per 10-20 ml. 50-100 mmHg negative pressure recovery was used immediately, and the filtered liquid was collected by a double layer sterile gauze into a disposable cup, centrifuged within 2 h after collection(3000 r/min × 10 min, 4 °C). The supernatant solution was ready for use.

### Outcome measures

Hemodynamics parameters (SBP, DBP, MAP, HR), SpO_2_ and respiratory rate were recorded when patients entered the operative room. Blood samples of arterial were taken 1 min before OLV(T_1_), 3 min after the completion of OLV(T_2_), and 30 min after T_2_(T_3_) for arterial blood gas analysis. Blood was collected through the internal jugular vein to prepare serum by centrifugation at T_1_, T_2_ and T_3_. Bronchoalveolar lavage was performed on the non-ventilated lung (RB5 or Li) at T_3_. Bronchoalveolar lavage fluid(BALF) was collected and centrifuged in time to get the supernatant. Serum MDA was measured by thiobarbituric acid method(kit purchased from Jiancheng Company of Nanjing, China) and the serum levels of SOD was determined by the Xanthine oxidase colorimetric method (kit purchased from Jiancheng Company of Nanjing, China).The concentrations of serum sICAM-1, TNF-α, NF-kB, IL-6, IL-10, SP-A,SP-B and SP-C were measured by ELISA (kit purchased from XiTang Company of Shanghai, China).

The diagnostic criteria for postoperative pulmonary complications(PPCs) include at least one of the following complications: respiratory infection, respiratory failure, hypoxemia, bronchospasm, atelectasis.The clinical outcome determined by PPCs was assessed as the secondary endpoint, and the postoperative hospital stay was recorded.

### Statistical analysis

According to the pre-experimental results, the sample size was calculated. Sample size was determined by NF-kB.The two groups were compared with independent t-test, with *p* < 0.05 as the standard for statistically significant, and missing rate of 0.1, on the basis of that, the number of estmated samples was 54 in total and 27 for each group. Values are median [25–75% Percentile], One-way ANOV (and nonparametric tests) was used for intra-group comparison, and Mann Whitney test was used in the comparison between groups, one-tailed *P* values. Numerical values were performed with Graphpad prism 6.0, *P* < 0.05 as statistically significant.

## Results

### Patient characteristics and surgical criteria

**Fig. 1:**
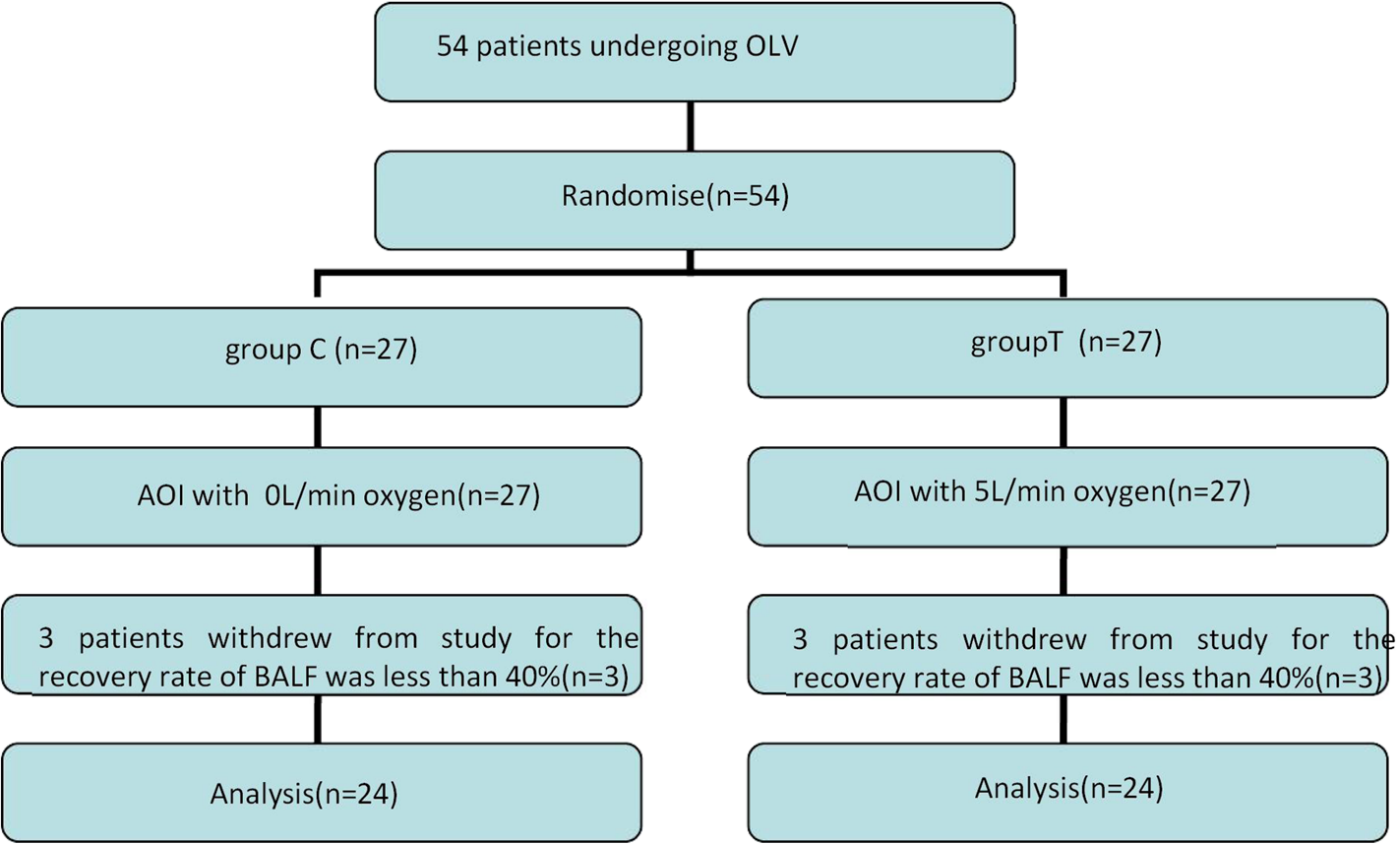
Patient enrollment flow diagram. This illustrates the flow of all patients screened, excluded, and randomized

Figure 1 presents the CONSORT diagram of patient recruitment. Among the 54 patients enrolled, 6 were excluded because of the recovery rate of BALF less than 40%. In total, 48 patients were statistically analyzed. None of these patients showed any signs of preoperative infection. There were no statistically significant differences in age, height, weight, sex, OLV time, operation time between the two groups (Table 1).

**Table 1:** Clinical Characteristics of Patients

	Group C	Group T	P
Age (years)	63[61.75–65.25]	63[59.75–65.25]	0.89
Height (cm)	166[162–170]	168[159.5–170.3]	0.41
Body weight (kg)	65[60–72.4]	65[60–73.5]	0.91
Male (%)	70.8	66.7	0.76
Durations of surgery (min)	238[199–306]	240[194–286]	0.58
Durations of OLV (min)	125[66–140]	100[80–134]	0.69
Fluid infusion volume (ml)	2000[1500–2075]	2000[1738–2275]	0.44
Urine volume (ml)	325[288–450]	400[295–562]	0.70

Clinical Characteristics of Patients, there had no significant difference between the groups. Values are median [25e-75e percentile] or constituent ratio(%), Mann Whitney test was used in the comparison between groups, two tailed *P* values

### Oxygenation index after OLV

**Fig. 2:**
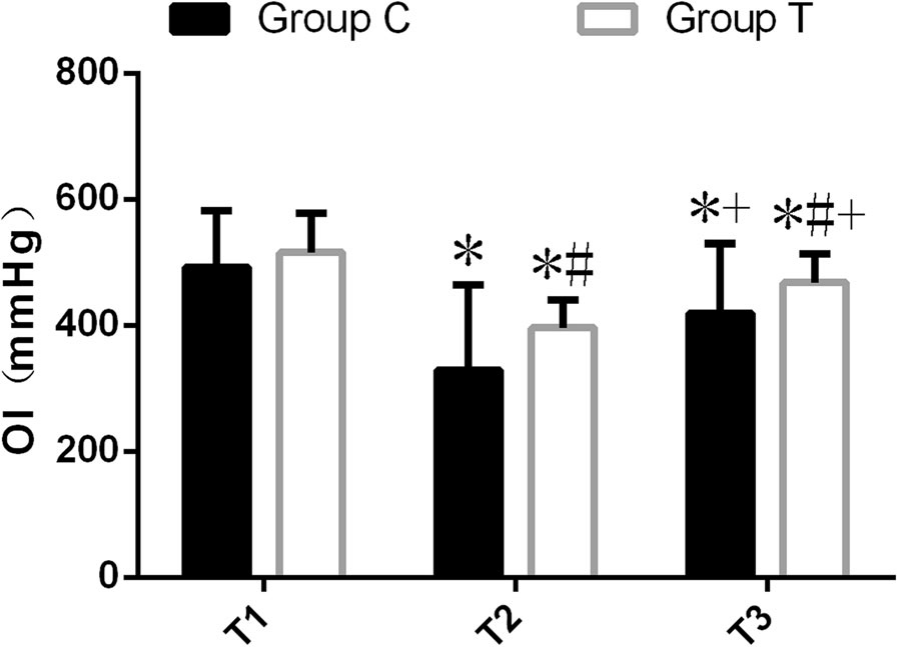
Comparison of oxygen Index at different time points(*n* = 24, $\overline{x}\pm s$) Compared with the same group at T_1_, ^*^P < 0.05; Compared with the same group at T_2_,^+^*P* < 0.05; Compared with group C, ^#^*P* < 0.05. One-way ANOVA (and nonparametric) was used for intra-group comparison, and Mann Whitney test was used in the comparison between groups, one-tailed *P* values

One lung ventilation resulted in an decrease in the measured of oxygenation index(OI) after OLV. OI at T_2_ and T_3_ of two groups were all decreased compared with those at T_1_ (*P* < 0.05); However, patients with AOI had better oxygenation in the recovery period, OI (394[367–426] and 478[440–497]mmHg, respectively) of group T at T_2_ and T_3_ were significantly higher than those (332[206–434] and 437[331–512]mmHg, respectively) of group C(*P* < 0.05) (Fig. 2).

Three patients in group C were forced to undergo two- lung ventilation due to deep desaturation, while none in group T.

### Inflammatory markers expression after OLV

One lung ventilation resulted in an increase in the measured inflammatory markers in both groups (calculated as difference in concentrations of inflammatory markers in serum, performed after OLV at T_3_ and before OLV at T_1_), however, the increase of inflammatory markers upon OLV in the group C was significantly higher than those of group T. Meanwhile, the inflammatory markers of group C was significantly higher than those of group T in BALF at T_3_. The serum levels of IL-10 of group T at T_3_ was increased significantly compared with those at T_1_(*P* < 0.05), and the IL-10 in both serum and BALF of group T were higher than those of group C at T_3_ (*P* < 0.05) (Tables 2 and 3).

**Table 2:** Changes of inflammatory markers of serum at each time point

		T1	T2	T3
NF-kB (pg/ml)	group C group T	30.67[17.57–39.82] 31.80[16.51–40.53]	35.04[22.92–56.49] 27.63[18.43–44.06]	42.82[23.86–67.84]^ab^ 30.19[21.89–43.86]^ac^
sICAM-1 (ng/ml)	group C group T	1.78[1.33–2.45] 1.77[1.55–2.03]	1.72[1.24–2.40] 1.89[1.45–2.11]	2.55[1.90–2.82]^ab^ 2.07[1.81–2.31]^abc^
TNF-α (pg/ml)	group C group T	24.74[(8.67–48.09] 26.61[12.62–33.12]	30.13[14.31–52.07] 30.01[24.00–34.37]	55.81[23.21–72.98]^ab^ 40.01[30.62–51.91]^abc^
IL-6 (pg/ml)	group C group T	14.74[8.96–22.14] 18.37[7.01–24.95]	27.19[13.66–46.24]^a^ 21.86[11.33–36.2]^a^	52.83[33.07–58.66]^ab^ 30.53[18.86–57.38]^abc^
IL-10 (pg/ml)	group C group T	1.91[1.43–2.65] 1.77[1.08–2.30]	1.75[1.20–2.93] 1.69[1.21–3.04]	1.50[1.01–2.10] 2.35[1.81–4.24]^ac^

Changes of inflammatory markers of serum at each time point,and the difference was statistically significant.Compared with those at T1,^a^*P* < 0.05;Compared with those at T2,^b^*P* < 0.05;Compared with those of group C,^c^*P* < 0.05.Values are median [25e-75e percentile],One-way ANOVA(and Nonparametric) was used for intra-group comparison, and Mann Whitney test was used in the comparison between groups,one-tailed *P* values

**Table 3:** Changes of inflammatory markers of BALF

	NF-kB(pg/ml)	sICAM-1(ng/ml)	IL-6(pg/ml)	IL-10(pg/ml)
Group C Group T	49.12[36.66–71.33] 33.35[17.11–52.50]^c^	0.59[0.22–0.71] 0.33[0.23–0.44]^c^	7.08[4.73–9.28] 3.67[2.15–6.5]^c^	1.91[0.97–2.40] 2.67[1.49–4.08]^c^

Changes of inflammatory markers of BALF. Compared with those of group C,

^c^*P* < 0.05.Values are median [25e-75e percentile], Mann Whitney test, two-tailed *P* values

### Surfactant protein A, B, C expression after OLV

**Fig. 3:**
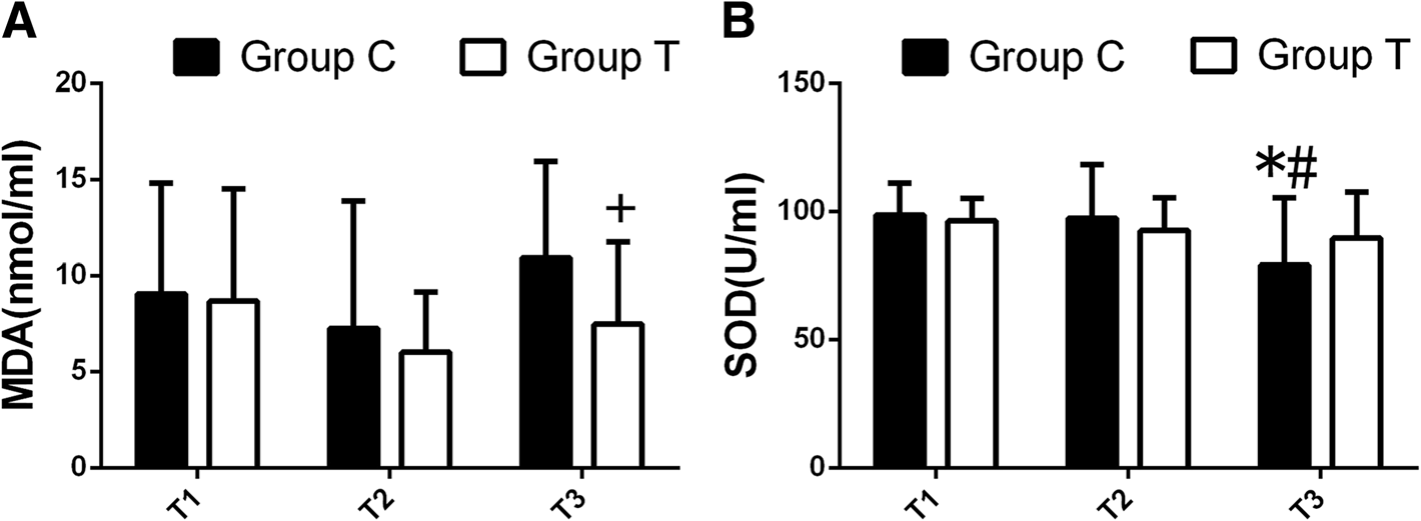
Concentration of MDA and SOD of serum (*n* = 24, $\overline{x}\pm s$). Concentration of MDA (**a**) and SOD (**b**) of serum,Compared with those at T1 ,*P < 0.05; Compared with those at T2 , ^#^*P* < 0.05; Compared with those of group C,^+^*P* < 0.05. One-way ANOVA(and nonparametric ) was used for intra-group comparison, and Mann Whitney test was used in the comparison between groups,one-tailed *P* values

One lung ventilation resulted in an increase in the measured surfactant protein A, B, C in serum of both groups (calculated as difference in concentrations of surfactant protein A, B, C in serum, performed after OLV at T_3_ and before OLV at T_1_). However, the levels of surfactant protein A, B, C of group T were lower than those of group C in serum at T_3_, and the results in BALF were the opposite (Table 4, Fig. 3).

**Table 4:** Changes of alveolar surfactant protein A, B, C of serum

		T_1_	T_2_	T_3_
SP-A (ng/ml)	group C	0.29[0.11–0.42]	0.38[0.22–0.52]^a^	0.45[0.34–0.61]^ab^
	group T	0.31[0.24–0.37]	0.38[0.31–0.47]^a^	0.39[0.32–0.47]^ac^
SP-B (ng/ml)	group C	4.19[3.05–6.03]	5.85[5.20–6.86]^a^	6.86[5.69–9.191]^a^
	group T	4.28[4.01–4.87]	4.80[4.63–5.39]^ac^	5.49[5.10–6.64]^abc^
SP-C (ng/ml)	group C	0.37[0.31–0.48]	0.66[0.45–0.80]^a^	1.06[0.59–1.73]^ab^
	group T	0.36[0.19–0.49]	0.47[0.32–0.61]^ac^	0.76[0.28–1.05]^abc^

Changes of alveolar surfactant protein A, B, C of serum at each time point, and the difference was statistically significant. Compared with those at T_1,_^a^ *P* < 0.05;Compared with those at T_2_, ^b^ *P* < 0.05;Compared with those of group C, ^c^ *P* < 0.05.Values are median [25e-75e percentile], One-way ANOVA (and nonparametric) was used for intra-group comparison, and Mann Whitney test was used in the comparison between groups,one-tailed *P* values

### Oxidative stress markers expression after OLV

One lung ventilation resulted in an decrease in the measured SOD in group C (calculated as difference in concentrations of SOD in serum, performed after OLV at T3 (83.73 [67.01–92.36]U/ml) and before OLV at T1(98.79[90.78–102.92]U/ml). Meanwhile, The BALF levels of SOD(23.88[14.70–33.93]U/ml) of group T were higher than those(15.99[10.33–24.16] U/ml) of group C (*p* < 0.05).

**Fig. 4:**
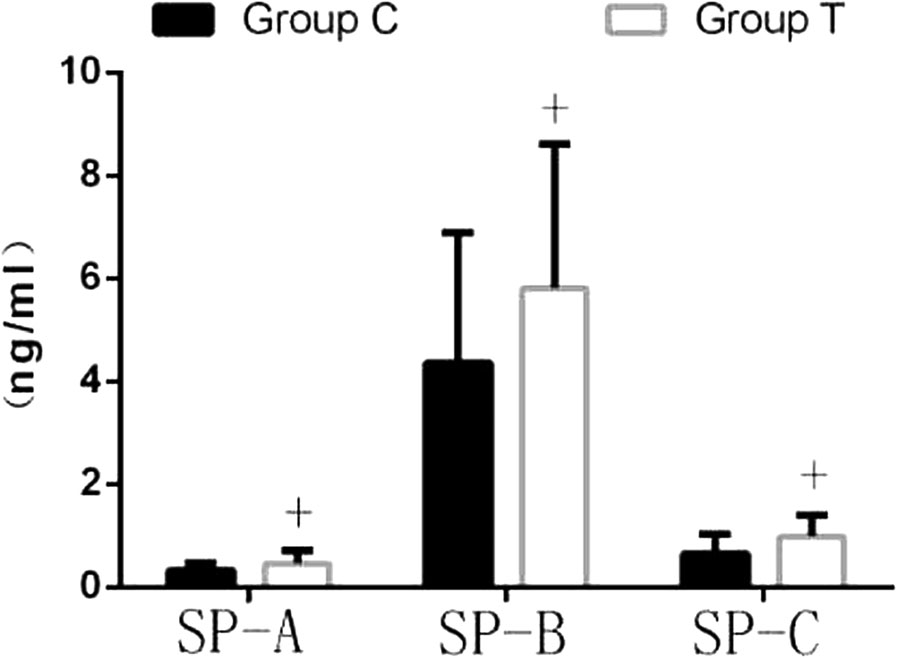
Comparison of alveolar surfactant protein A ,B, C of BALF. (*n* = 24, $\overline{x}\pm s$) Concentration of alveolar surfactant protein A, B, C of BALF. Compared with those of group C, ^+^*P* < 0.05. (Mann Whitney test,one-tailed *P* values)

**Fig. 5:**
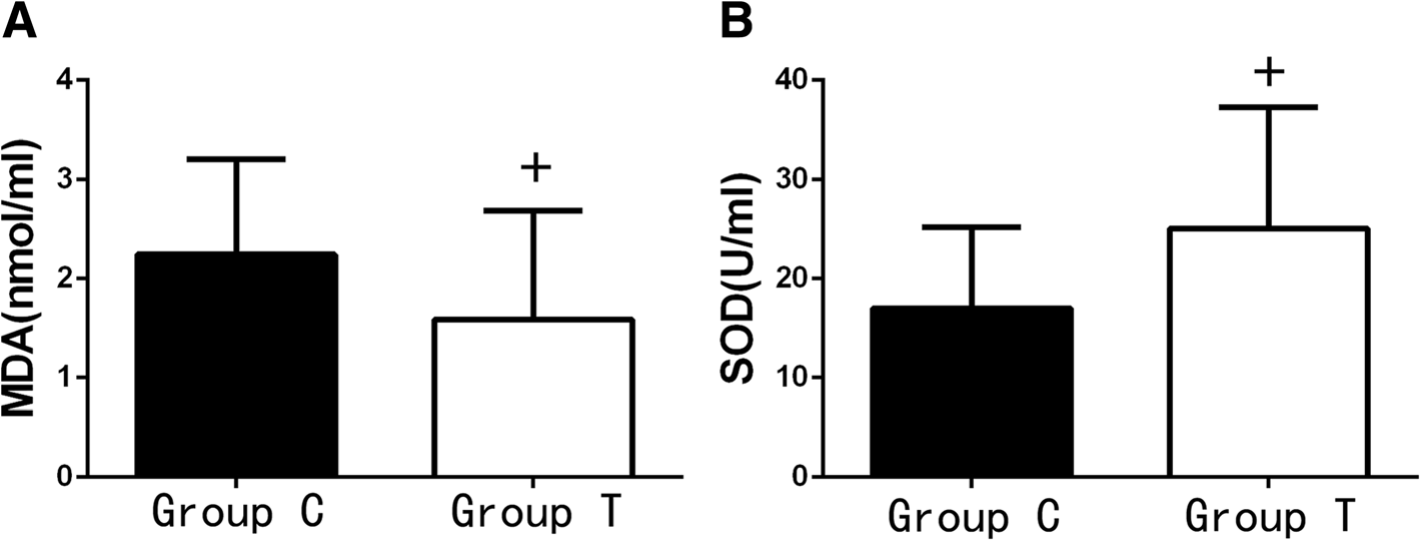
Concentration of MDA (**a**) and SOD (**b**) of BALF. The BALF levels of MDA (2.25 ± 0.96 and 1.59 ± 1.10 nmol/ml), SOD (17.02 ± 8.17 and 25.10 ± 12.18 U/ml) at T_3_ of group C and T, respectively, and the difference was statistically significant (Mann Whitney test, one-tailed *P* values). Compared with group C, ^+^*P* < 0.05

The levels of MDA in both serum and BALF of group T (8.60[4.14–9.85] and 1.88[1.33–3.08]nmol/ml, respectively) at T_3_ were all significantly lower than those of group C(11.10[6.57–13.75] and 1.280[1.01–1.83] nmol/ml)(*p* < 0.05) (Figs. 4, 5).

### Clinical evaluation

Further studies have shown that there was no statistical difference between the two groups in terms of postoperative hospital stay and the incidence of PPCs (Table 5).

**Table 5:** Comparison of the incidence of PPCs and postoperative hospitalization time

	Postoperative hospital stay (days, median [25e-75e percentile])	Incidence of PPCs (occurrence / total sample number)
group C	11[11–13]	3/24
group T	12.5[11–14]	2/24
P	0.96	0.64

Comparison of the incidence of PPCs and postoperative hospitalization time, there was no statistical difference between the two groups.(Mann Whitney test, two-tailed P values or X^2^ test

## Discussion

Lung protective ventilation strategy(LPVS) mainly includes low tidal volume ventilation+PEEP, combined with some recruitment maneuvers. A large number of studies have shown that LPVS can significantly reduce the release of inflammatory cytokines, and also reduce ventilator-associated atelectasis and lung injury induced by mechanical ventilation, while avoiding excessive expansion and collapse of the lung [10–12]. However, in recent years, some studies have shown that the application of LPVS during OLV does not show the advantages above [13], their results indicated that mechanical ventilation with high VT and no PEEP did not result in higher cytokine levels when compared with strategies including a reduction of VT associated with PEEP during major surgical procedures. Based on the literature above and the fact that the best PEEP is difficult to determine, the respiratory parameters we used in this clinical trial are as follows: tidal volume(VT)8-10 ml/kg(Mean 8.2 ml/kg) during TLV and 5-6 ml/kg(Mean 5.3 ml/kg) during OLV + ZEEP, combined with some recruitment maneuvers every 30 min, and maintain peak airway pressure less than 25cmH_2_O during ventilation to avoid lung injury caused by high tidal volume. It can also prevent excessive collapse of the lung and the occurrence of atelectasis.

Michelet P et al [14] reported the decrease of oxygenation function and the increase of intrapulmonary shunt rate in patients with esophageal cancer during and 1 h after OLV. Alveolar hypoxia induces inflammation and the use of AOI to maintain a certain swelling of the lung can delay the occurrence of lung injury [15]. After using AOI, even during the period of lung collapse, some of the alveoli remain unobstructed with the proximal airway, increasing the non-ventilated side oxygen concentration [16], facilitating gas exchange and reducing pulmonary shunt during OLV [17].The results of this study suggest that Patients of both groups have varying degrees of decline in oxygenation function after OLV, which is consistent with previous literature. The AOI strategy resulted in better oxygenation preservation in group T, indicating that the AOI can improve the oxygenation of the lung after OLV. In addition, it also can effectively reduce the occurrence of hypoxia after OLV, and play an effective protection role on lung tissue.

Michelet P et al [14] also reported that esophagectomy induces a systemic inflammatory response whose extent has been recognized as a predictive factor of postoperative respiratory morbidity. You Z [18] showed that the expression of NF-kB,TNF-α and sICAM-1 in rabbit lung tissue increased after OLV, the use of NF-kB inhibitor can reduce the expression of TNF-α, sICAM-1, and alleviate lung injury. The literature indicated that the increased expression of cytokines and chemokines caused by the activation of NF-kB is an important feature of inflammation. In the current clinical trial, the changes in cytokine level that we observed in the group C were in accord with previous studies [14, 19], the production of the corresponding inflammatory mediators NF-kB, TNF-α, sICAM-1 in the respiratory compartment was quantitatively assessed upon OLV for esophagectomy, the increase of these mediators is diminished in group T compared with group C. These results indicated that OLV can activate the NF-kB signal transduction pathway and up-regulate the expression of TNF-α, sICAM-1, IL-6, a significantly more pronounced inflammatory reaction is present in the group C, AOI might have the potential to delay or preclude the resolution of the systemic inflammatory to the OLV for esophagectomy.

The secretion or exudation of bronchoalveolar which is close to the parenchyma is almost free from external interference.BALF can accurately reflect the pathological changes in the lung [20], such as the regional inflammation induced by OLV includes both lungs. Sugasawa et al. found that the pulmonary inflammatory response in the ventilated lung(such as IL-1,IL-6 and IL-8 increase) was significantly stronger than the non-ventilated lung [21]. Zingg also observed that release of inflammatory substances in the ventilated lung were more pronounced [22]. In this study, we only performed bronchoalveolar lavage on the non-ventilated lung, and found that the inflammatory markers such as NF-kB, TNF-α, sICAM-1,IL-6 of group C was significantly higher than those of group in BALF. These results also indicated that OLV can activate the regional inflammation induced by OLV, AOI might have the potential to delay or preclude the resolution of the regional inflammatory response of non-ventilated lung to the OLV for esophagectomy.

Recent studies have shown that pulmonary surfactant is a multifunctional complex located on the surface of alveolar epithelial cells, which is composed of key lipid proteins [23]. SP-A is considered to be an important regulator of inflammation/anti-inflammatory system [24]; SP-B is responsible for resistance to surface tension and prevention of collapse of pulmonary alveoli [25, 26]. SP-C can reduce the surface tension of alveoli, increase lung compliance, avoid the collapse of alveolar transition, and has the function of local defense [27]. In the current clinical trial, we first demonstrated that the level of serum alveolar surfactant protein A, B, C in both groups was increased after OLV, but that in group T was significantly lower than that in group C, while the contents of alveolar surfactant protein A, B, C in BALF of group T were significantly higher than those of group C. These results indicated that AOI can effectively reduce the loss of alveolar surfactant protein A, B, C in the non-ventilated lung.

The mechanism of OLV inducing lung injury is very complicated, including mechanical ventilation, hypoxemia and oxidative stress response [28, 29]. It is generally accepted that lung cancer patients are more sensitive to oxidative stress [30] and the generation of reactive oxygen species (ROS) and the release of proinflammatory cytokines play an important role in the lung injury [31].The study showed that after OLV switched to TLV, the lung tissue produced a certain amount of oxygen free radicals [32]. Therefore, the patients selected in this study were esophageal or cardiac cancer to avoid the interference of surgical operations, and we first observed the changes of SOD and MDA in serum before and after OLV, the results indicated that hypoxia/reperfusion could aggravate oxidative stress response with the recovery of TLV, while the oxidative/antioxidant system was out of balance, and AOI can alleviate the oxidative stress response by improving oxygenation function of non-ventilated lung.

Some scholars were worried about the atelactasis induced by the pure oxygen in the alveoli which replaced the nitrogen after AOI. We found that the incidence of atelectasis did not increase after AOI, which may be related to decreased collapsed alveoli, reduced loss of alveolar surface protein B, C and alliviated pulmonary inflammation.

Although we initially observed the effects of AOI on postoperative hospital stay and PPCs, but the results have not demonstrated significant difference between groups. Four factors need to be considered as possible triggers for the results:(1) The surgeon’s persistent work habits affect the postoperative hospital stay. If the patient does not have serious postoperative complications, such as anastomotic fistula, incision infection, PPCS and so on, the patient was discharged about a fixed time after surgery. (2) The regional inflammation induced by OLV includes both lungs. In this study, we only performed bronchoalveolar lavage on the non-ventilated lung, and only observed the regional inflammation and oxidative stress of the non-ventilated lung. The contralateral lung injury and its effect on systemic inflammation need further study. (3) The use of antibiotics after surgery interfereed with the observation of PPCS. (4) The sample size in this study is small, and there may be bias in observing the incidence of PPCs, so we need to expand the sample size for further study. However, this study was not powered for clinical endpoints, and further studies should be performed to assess the influence of such a strategy of AOI on clinical outcomes.

## Conclusions

AOI with 5 L/min oxygen on non-ventilated lung during OLV can improve the oxygenation function after OLV, and relieve the inflammation and oxidative stress responses in the systemic and the non-ventilated lung after OLV associated with esophagectomy.
